# Percutaneous closure of a paravalvular leak from a prosthetic mitral valve dehiscence in a young high-risk patient: case report

**DOI:** 10.1093/ehjcr/ytac242

**Published:** 2022-06-16

**Authors:** Ozge Ozden, Hatice Kemal, Gülsüm Bingöl, Ömer Göktekin

**Affiliations:** Cardiology Department, Memorial Bahçelievler Hospital, Bahçelievler Merkez, Adnan Kahveci Blv. No. 227, 34180 Bahçelievler, İstanbul, Turkey; Cardiology Department, Near East University Hospital, Nicosia, Turkish Republic of Northern Cyprus; Cardiology Department, Memorial Bahçelievler Hospital, Bahçelievler Merkez, Adnan Kahveci Blv. No. 227, 34180 Bahçelievler, İstanbul, Turkey; Cardiology Department, Memorial Bahçelievler Hospital, Bahçelievler Merkez, Adnan Kahveci Blv. No. 227, 34180 Bahçelievler, İstanbul, Turkey

**Keywords:** Prosthetic mitral valve, Paravalvular leak, Percutaneous device closure, 3D echocardiography

## Abstract

**Background:**

Paravalvular leak (PVL) is a common, serious complication related with prosthetic valve replacement. Although surgical closure reoperation is the choice of treatment, percutaneous device closure is a good alternative with good results in patients with very high surgical risk.

**Case summary:**

In this case report, we present the percutaneous closure of PVL of mitral valve replacement (MVR), in a patient with cardiogenic shock who failed conservative medical treatment and was deemed inoperable due to recurrent operations. Successful closure of the PVL with the use of the four consecutive PVL closure devices was performed under general anaesthesia with guidance of 2D and 3D transoesophageal echocardiography. The procedure was performed with no complications and the patient has remained asymptomatic after 10 months following the PVL closure procedure with marked improvement in her NYHA class and echocardiographic values.

**Discussion:**

Percutaneous PVL closure is a very challenging and high clinical skills requiring procedure, but has a good success and low complication rate in high-risk patients. It is not a standard procedure and the type and size of device should be tailored for each patient with a good 2D and 3D echocardiographic guidance.

**Learning points**

- Percutaneous closure represents a safe and feasible option; this can be a valuable option in high- or prohibitive-risk surgical candidates.
- A good 3D TOE guidance is of utmost importance to determine the success of the PVL closure procedure.

## Introduction

Paravalvular leak (PVL) is a common complication after valvular replacement, with reported incidences at follow-up of 7–17% in mitral position.^1^ Almost 1–3% patients with PVL require reoperation due to symptoms of congestion, haemolysis, or in most cases both.^2^ Surgical correction is the first-line recommended treatment but reoperation has high mortality and morbidity rates. Percutaneous PVL closure is an alternative treatment approach but every case is unique and the treatment approach is always patient tailored.

## Timeline

**Table:** 

10/10/2020	Minimal invasive mitral valve replacement (MVR)
3/1/2021	Hospital admission; septic shock, intubated for ARDS. TOE revealed massive vegetation and valvular dehiscence of the prosthetic mitral valve. Urgent redo MVR was performed on the same day
1/4/2021	Hospital readmission; acute pulmonary oedema. Paravalvular leak (PVL) seen on TOE.
30/4/2021	The patient had a successful PVL closure procedure with 4 closure devices and her functional status improved from NYHA IV to NYHA II afterwards.
3/2/2022	The patient has NYHA class II functional status with good echocardiographic results.

## Case presentation

A 45-year-old female with surgical prosthetic MVR 4 months ago was hospitalized with septic shock and intubated immediately due to acute respiratory distress syndrome. Transoesophageal echocardiography (TOE) revealed massive vegetation with abscess formation on the posterior commissure of the prosthetic valve and dehiscence. The patient underwent very high-risk urgent operation on the same day. The mitral valve was replaced with 21 mm bileaflet mechanical prosthetic valve (St Jude Medical, Inc.; St Paul, MN, USA) and mitral annulus was reconstructed using pericardium with no residual PVL.

**Figure 1:**
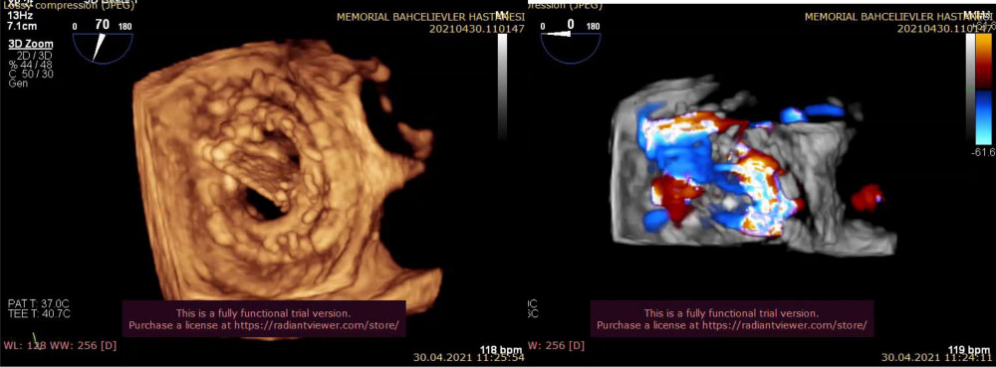
The dehiscence of the prosthetic valve and the paravalvular regurgitation with colour Doppler.

**Figure 2:**
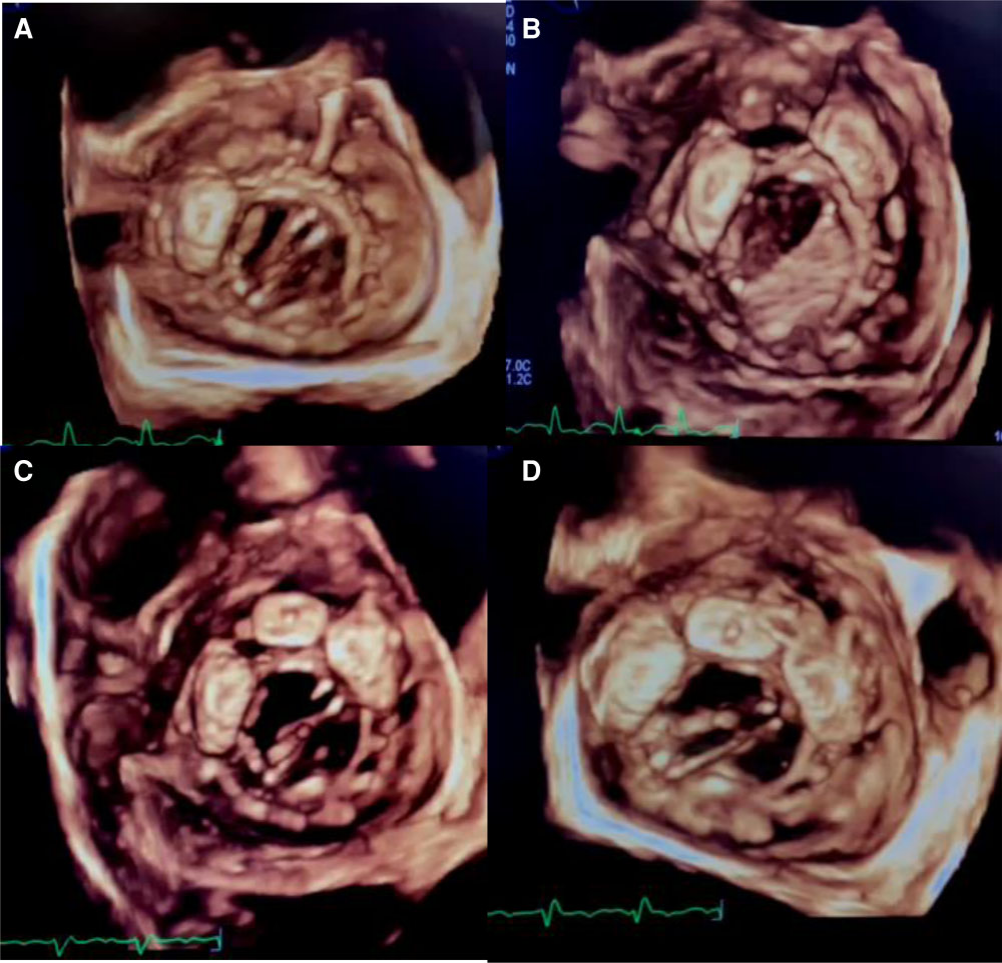
Step-by-step the deployment of closure Device 1 (*A*), Device 2 (*B*), Device 3 (*C*), and a duct occluder (*D*). Supplementary material online, *Video S1*. Transoesophageal echocardiography showing the dehiscence of the prosthetic valve and the paravalvular regurgitation with colour Doppler. Supplementary material online, *Video S2*. Intraprocedural transoesophageal echocardiography showing the closure devices and colour Doppler imaging at the end of procedure.

After discharge with no complication, the patient was rehospitalized on the postoperative third month with acute pulmonary oedema. At admission, the patient was orthopnoeic and anxious. Her blood pressure was 90/60 mmHg, her rhythm was atrial flutter with 2:1 AV block and her SpO_2_ was 85% in room air. She had no markers of inflammation or infection. She had a haemolytic anaemia due to PVL. The patient was evaluated with 2D and 3D TOE, and a dehiscence was demonstrated by 3D TOE between 10 and 3 o'clock position, which makes approximately 50% of the mitral annulus, and this was also confirmed by 3D colour Doppler imaging (*Figure 1*). Resurgery was considered as very high risk by the heart team and percutaneous PVL closure was planned. First device was a 14 mm Amplatzer™ Vascular Plug (AVP) III (St Jude Medical) and it was implanted at 10 o’clock position. After the first device implantation, the remaining degree of PVL was moderate. To close the remaining leak, second same device was placed at 1 o’clock position. A third device was implanted between the two devices to close the residual mild-moderate PVL. The residual mild PVL between the second and third devices was completely closed with the 6 mm Amplatzer™ Duct Occluder II (St Jude Medical) and there was no residual PVL at the end of the procedure (*Figure 2*). Her functional capacity improved from NYHA Class IV to Class II at the end of the first week.

At 10 months of follow-up, the patient has NYHA Class II functional capacity and she is on a metoprolol 50 mg 2 × 1, ramipril 5 mg 1 × 1, furosemid 40 mg 2 × 1 treatments. She has no peripheral oedema and her laboratory findings are totally normal.

## Discussion

The PVL can cause serious clinical outcomes in about 1–5% of patients which include symptomatic congestive heart failure, severe haemolytic anaemia, and endocarditis.^3^ Our patient presented with cardiogenic shock. While cardiogenic shock is a very high mortality condition in itself, reoperation for PVL would greatly increase the existing mortality risk. Percutaneous closure of PVLs has emerged as an alternative procedure to cardiac surgery, particularly in patients who have undergone multiple previous valvular operations and have high mortality and morbidity risk.^4^

The PVL closure is a complex and technically demanding procedure, as PVLs varies in size, shape, and complexity of the defect. Cardiac imaging plays great role and clinical experience is essential, since it is not a common procedure.^5^ A live 3D TOE is mandatory for guiding the procedure. The interventionist is dependent on the live 3D TOE while crossing the PVL, determining the type and size of the device, deploying of the device, and assessing the mechanical valve function after leak closure. The use of 3D TOE has led to improvement in accurate delivery and deployment of devices for mitral PVLs.

One of the challenging points of this case was the big defect accounting more than 50% of the anterior annulus of the mitral valve and the number of closure devices used. The AVPs are the most commonly used occluder devices and especially the oblong AVP III has a clear advantage over other occluders with a circular cross-section. We had a patient tailored approach, step-by-step monitoring residual PVL with 3D TOE, 3 AVP III was used and a duct occluder for the final residual leak, resulting in a good success. Clinical experience in treating these high-risk patients plays vital role as selecting correct device size and deploying correctly are vitally important.

## Supplementary Material

ytac242_Supplementary_Data

## Lead author biography

Dr Özge Özden is a cardiologist at Memorial Bahçelievler Hospital in İstanbul/Turkey. She graduated Istanbul University Cerrahpaşa Medical Faculty in 2009 as first ranking student and received her specialization at the same university in 2013. Her current duties are EACVI Web & Communication Committee Member (2020–2022), EACVI HIT Committee Member (2020–2022), and ESC Communication Committee Member (2020–2022). She is a multimodality imaging expert and was awarded with EACVI Training grant on CMR in 2017. She trained CMR at Bristol Heart Institute under supervision of Dr. Chiara Bucciarelli-Ducci in 2019. She was also trained in cardiac CT, 3D echocardiography, interventional echocardiography and structural heart disease imaging at different centers. She was also one of the previous EACVI HIT ambassadors of Turkey for CV Imaging.

## Supplementary material

**Consent:** The authors confirm that written consent for submission and publication of this case report including images and associated text has been obtained from the patient in line with COPE guidance.

**Funding:** None declared.

### Data availability

The data underlying this article are available in the article and in its online supplementary material.
