# Supplementary figures and images for: Percutaneous closure of a paravalvular leak from a prosthetic mitral valve dehiscence in a young high-risk patient: case report
Source: Eur Heart J Case Rep. 2022 Jun 16;6(7):ytac242. doi: 10.1093/ehjcr/ytac242 (PMC9290529; doi:10.1093/ehjcr/ytac242)

## Slide 1
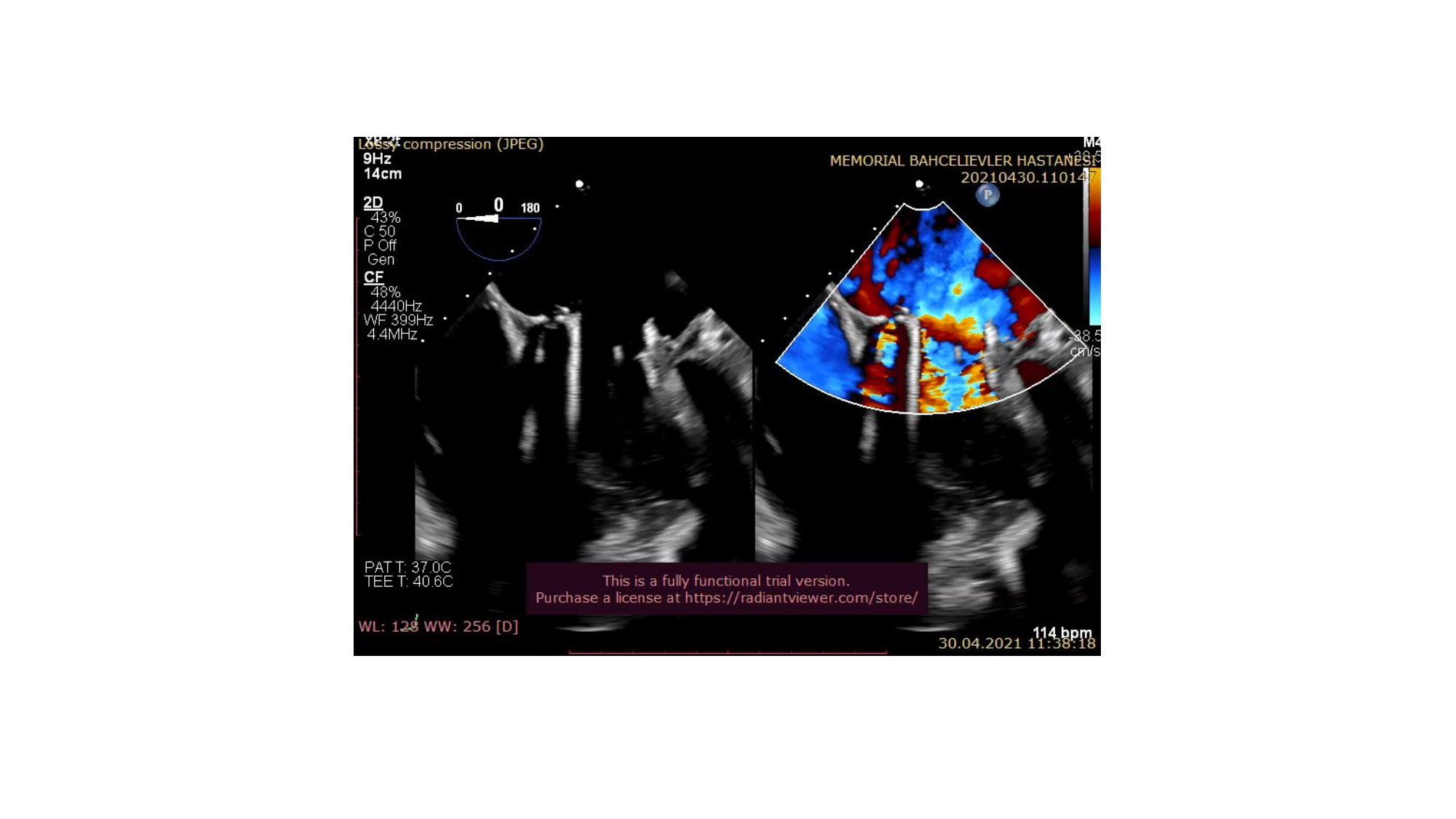

#

## Slide 2
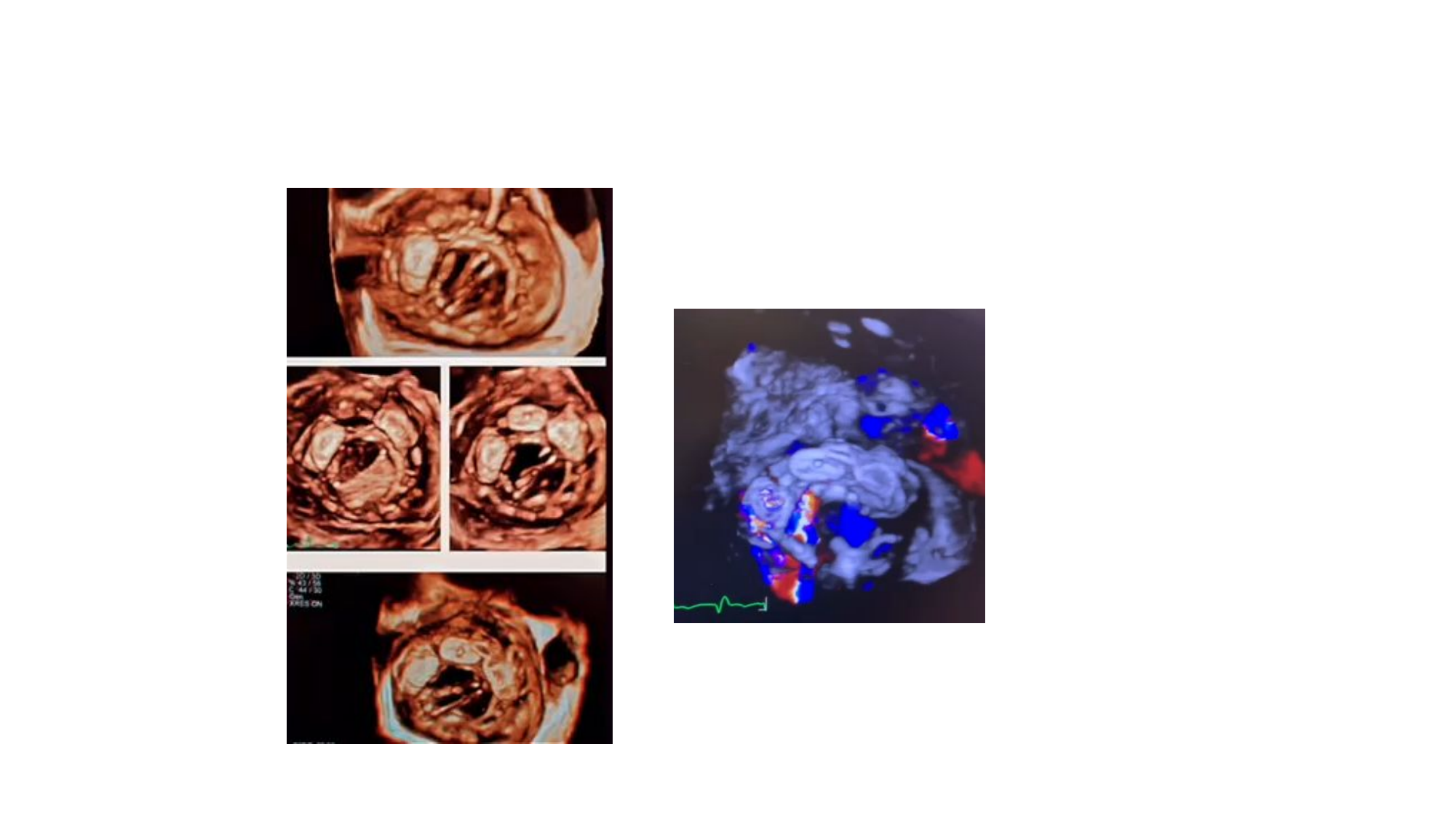

Supplement: ytac242_Supplementary_Data [file ytac242_supplementary_data.pptx]
